# Hip and Wrist-Worn Accelerometer Data Analysis for Toddler Activities

**DOI:** 10.3390/ijerph16142598

**Published:** 2019-07-21

**Authors:** Soyang Kwon, Patricia Zavos, Katherine Nickele, Albert Sugianto, Mark V. Albert

**Affiliations:** 1 Stanley Manne Children’s Research Institute, Ann & Robert H. Lurie Children’s Hospital of Chicago, Chicago, IL 60611, USA; 2 Department of Computer Science, Loyola University Chicago, Chicago, IL 60660, USA

**Keywords:** physical activity, sedentary behavior, young children, activity recognition, activity classifier, machine learning

## Abstract

Although accelerometry data are widely utilized to estimate physical activity and sedentary behavior among children age 3 years or older, for toddlers age 1 and 2 year(s), accelerometry data recorded during such behaviors have been far less examined. In particular, toddler’s unique behaviors, such as riding in a stroller or being carried by an adult, have not yet been examined. The objective of this study was to describe accelerometry signal outputs recorded during participation in nine types of behaviors (i.e., running, walking, climbing up/down, crawling, riding a ride-on toy, standing, sitting, riding in a stroller/wagon, and being carried by an adult) among toddlers. Twenty-four toddlers aged 13 to 35 months (50% girls) performed various prescribed behaviors during free play in a commercial indoor playroom while wearing ActiGraph wGT3X-BT accelerometers on a hip and a wrist. Participants’ performances were video-recorded. Based on the video data, accelerometer data were annotated with behavior labels to examine accelerometry signal outputs while performing the nine types of behaviors. Accelerometer data collected during 664 behavior assessments from the 21 participants were used for analysis. Hip vertical axis counts for walking were low (median = 49 counts/5 s). They were significantly lower than those recorded while a toddler was “carried” by an adult (median = 144 counts/5 s; *p* < 0.01). While standing, sitting, and riding in a stroller, very low hip vertical axis counts were registered (median ≤ 5 counts/5 s). Although wrist vertical axis and vector magnitude counts for “carried” were not higher than those for walking, they were higher than the cut-points for sedentary behaviors. Using various accelerometry signal features, machine learning techniques showed 89% accuracy to differentiate the “carried” behavior from ambulatory movements such as running, walking, crawling, and climbing. In conclusion, hip vertical axis counts alone may be unable to capture walking as physical activity and “carried” as sedentary behavior among toddlers. Machine learning techniques that utilize additional accelerometry signal features could help to recognize behavior types, especially to differentiate being “carried” from ambulatory movements.

## 1. Introduction

Despite the recognized health benefits of physical activity [1], an inactive lifestyle is common among children [2]. Children who were inactive at age 5 years tend to follow an inactive lifestyle trajectory throughout childhood and adolescence [2]. Some evidence suggests that a substantial proportion of preschoolers aged 3 to 5 years old engage in physical activity lower than the recommended level [3,4,5]. It appears that an understanding of when and how a habit of physical inactivity develops requires investigation starting at a younger age [6]. However, only a few studies [7,8,9,10,11,12,13] have investigated physical activity levels for children under age 3 (e.g., toddlers aged 1 and 2 years), partly because of a significant methodological gap regarding physical activity assessment for that age group, particularly related to accelerometer data processing. A triaxial accelerometer is the most widely accepted device to measure physical activity and sedentary behavior in children as well as adults.

To estimate physical activity and sedentary behavior among toddlers, previous studies have proposed intensity-based (i.e., sedentary, light-intensity activity, and moderate- and vigorous-intensity activity) ActiGraph accelerometer vertical axis count cut-points [14,15]. Recent studies have also utilized machine learning techniques to classify physical activity behaviors. The machine learning techniques are advanced analytic techniques that can capture complex dependencies and non-linearities in abundant raw accelerometer data to improve recognition of physical activity [16]. The Nam and Park study [17] has developed machine learning activity classifiers to recognize toddler’s behaviors, such as walking and crawling. A few preschooler studies [18,19,20] also developed machine learning activity classifiers to classify activity intensities and/or behaviors. However, these cut-points and machine learning classifiers were developed without consideration of toddler’s unique activities, such as being carried by an adult and riding in a stroller/wagon.

While a toddler is “carried” by an adult, a significant amount of acceleration signals could be recorded, reflecting the ambulatory movements of the adult. For example, if high vertical accelerometer signals are recorded during the “carried” behavior, use of the vertical axis cut-point could misclassify the “carried” behavior, which is a type of sedentary behavior, as physical activity. Similarly, signals recorded while riding in a stroller/wagon should be recognized as sitting or sedentary behavior. However, little has been documented about the accelerometry signal outputs recorded during toddlers’ various types of behaviors, particularly during these unique behaviors. The objective of this study was to describe accelerometry signal outputs recorded during toddlers’ participation in nine types of behaviors (running, walking, climbing up/down, crawling, riding a ride-on toy, standing, sitting, riding in a stroller/wagon, and being carried by an adult). We utilized both raw acceleration (actual g-force; sub-aim one) [21] and activity count data (sub-aim two). We explored whether machine learning techniques could help differentiate being “carried” from ambulatory behaviors, such as running, walking, climbing up/down, and crawling (sub-aim three). This report will provide knowledge to refine the accelerometer data processing algorithms for toddlers, such as activity count cut-points or machine learning classifiers.

## 2. Materials and Methods

### 2.1. Participants

In 2018, 24 toddlers aged 13 to 35 months (50% girls; 50% one-year olds) were recruited among visitors of a commercial indoor child playroom (1500 square-foot open play space) located in Chicago, USA. The eligibility criteria included age between 13 and 35 months and being able to independently walk. A $30 gift card was given to each participant for their time and effort. Parents of the participating children provided written informed consent. This study was approved by the Institutional Review Board of Ann & Robert H. Lurie Children’s Hospital of Chicago.

### 2.2. Data Collection

A previous validation study [16] has demonstrated that free-living data outperform lab data in capturing variations in movement timing, types, and frequency in everyday life. Therefore, we conducted the activity trials in the commercial playroom that participants were familiar with, rather than a research lab, to offer participants a setting in which they could more naturally engage in physical activity. ActiGraph wGT3X-BT accelerometers (ActiGraph Inc; Pensacola, FL, USA) were used to collect acceleration data at 30 Hz (range ±6 g). Participants were fitted with two ActiGraph GT3X-BT accelerometers, one on the hip and the other on the non-dominant wrist or the left wrist when the dominant hand was unknown (*n* = 22 on the left wrist). Participants were encouraged by their caregivers to perform the nine behaviors, while engaging in different activities. Example activities included a race for running, walking up the stairs of a slide for climbing, kitchen play for standing, going through a tunnel for crawling, and block play for sitting. In addition, participants were carried by their caregivers and pushed in a stroller/wagon. Participants’ performances were video-recorded using a GoPro Hero5 Black camera. The participants completed the activity trial in an average of 15 min per participant, with a range from 8 min to 25 min.

### 2.3. Behavior Labeling

The video data were downloaded and used for behavior labeling. We found that one video file that contained the second half of the activity trial for one participant was corrupted and unable to be used for behavior labeling. Three co-authors (SK, KN, and PZ) created a coding scheme for the nine behaviors of interest (“run,” “walk,” “climb” up/down, “crawl,” ride a “ride-on toy,” “stand,” “sit,” ride in a “stroller”/wagon, and being “carried” by an adult), as well as for other unprescribed behaviors performed by toddlers, such as transitional movements, standing and moving, bouncing/jumping, and sliding down a slide. The behavior definitions are briefly described in Table 1. Three coders (SK, KN and PZ) independently coded the first four participants’ videos. Next, the coded data (behavior label, behavior start time and end time in seconds) were compared between the coders to identify any disagreements. We reviewed the video images that were associated with the disagreements, discussed, resolved all disagreements, and updated the coding scheme as needed. Based on the updated coding scheme, KN and PZ independently coded the next 10 participants’ videos, and SK and PZ independently coded the remaining 10 participants’ videos. Of 3308 coded behaviors from the 20 participants’ videos, 3175 (96%) were concordant between the two coders. All disagreements between the coders were again resolved using the same procedure described above. We found that the disagreements were mostly due to confusion between “standing and moving” and “transition to walking” or between “standing still” and “standing and moving”. In the end, a total of 4167 behaviors were labeled. For the current study, we excluded the following data. First, we excluded the data from three participants: one participant (303 behavior labels) who was found in the video review to be wearing the waist belt too high on the trunk and frequently taking off and resisting putting the wrist band back on, and two participants (535 behavior labels) whose video clock times were unable to be verified for accuracy. Second, of the 3229 behavior labels coded from the 21 participants, we only extracted the nine behavior labels of interest that were performed for at least five consecutive seconds, which resulted in 664 behavior labels (on average, 74 labels per behavior and 32 behavior labels per participant).

### 2.4. Accelerometer Data Processing

ActiGraph accelerometer data were extracted using ActiLife software version 6.13. The extracted accelerometer data were synchronized with the video-based behavior label data based on the video and accelerometer time stamps in seconds. To ensure the accuracy of the synchronization, we visually inspected the accelerometer signal magnitude for walking/running (active; large accelerometer signals expected) and sitting/standing still (inactive; small accelerometer signals expected) annotations over the entire trial time (*x*) in graphs for each participant. The accelerometer data for the first and the last second of the behavior label were excluded to ensure that only accelerometer data during a 100% single behavior, not during potential transitional behaviors, were included. For example, if “walk” was labeled from 10:00:00 to 10:00:05, only the accelerometer data between 10:00:01 and 10:00:04 were used, excluding those at 10:00:01 and 10:00:05.

To describe the accelerometry signals during the nine behaviors, we used four activity count measures: vertical (axis one), horizontal (axis two), and perpendicular (axis three) axis and vector magnitude [√(*x*^2^ + *y*^2^ + *z*^2^)] counts for each of the hip and wrist data. Axes one, two, and three counts are the count data generated by the ActiLife software (proprietary information). We calculated vector magnitude based on axes one, two, and three counts. Although orientation to gravity across the three axes would change based on the position of the child, vector magnitude is not affected by the orientation because vector magnitude is the sum of the three-axis movement magnitude. For each of the four measures, the sum per behavior and participant was calculated. The sum was divided by total time (seconds) for that specific behavior and multiplied by five to obtain the measures per 5 s (5 s).

The data processing described above revealed that “carried,” a type of sedentary behavior, tended to present higher vertical axis counts than “walk.” To explore whether utilization of various accelerometry signal features could help to differentiate “carried” from “ambulation”, such as “run,” “walk,” “crawl,” and “climb,” we performed the following data processing. Raw accelerometry data (g) were segmented into non-overlapping five-second windows. Only windows describing a 100% single behavior (“carried,” “run,” “walk,” “crawl,” or “climb”) were selected. For each accelerometer data window, 30 time-domain and 48 frequency-domain features were extracted. For time-domain variables, in addition to horizontal (*x*), vertical (*y*), and perpendicular (*z*) axes, vector magnitude [√(*x*^2^ + *y*^2^ + *z*^2^)] and cross-correlation (*xy*, *yz*, *xy*) variables were used. Time-domain features selected included mean, standard deviation (SD), median, minimum (min), maximum (max), skewness, and kurtosis of *x*, *y*, and *z*, mean, median, min, max, skewness, and kurtosis of vector magnitude, and mean of the cross-correlation variables. To convert the signal data into the frequency domain, we used the fast Fourier transform (FFT) algorithm to quantify the frequency of periodic motion in a signal. Frequency-domain features selected included mean, SD, min, max, weighted mean, min, and max, and the first 10 bins of FFT-based *x*, *y*, and *z* signals. To construct a classification model for two behavior categories (“carried” vs. “ambulation”), we used a random forest classifier that has been shown to have higher performance for activity recognition [18,22]. A feature selection step was not used as the machine learning model implicitly selected features through hyperparameter tuning. Grid search cross-validation was performed using 10-fold cross-validation over the entire data set to identify hyperparameters. Classifier performance was evaluated using a leave one-subject-out (LOSO) cross-validation method. All machine learning analysis was conducted using the Scikit-Learn machine learning package for Python 3.7 (http://scikit-learn.org).

### 2.5. Statistical Analysis

Descriptive analyses, including distribution analyses, were conducted for three axis and vector magnitude count variables. Mann-Whitney tests were conducted to compare the medians of vertical axis and vector magnitude counts between any two behaviors. To explore whether the lower hip vertical counts for “walk” are associated with shorter stature (indicated by age), we compared the median hip vertical counts between one-year olds and two-year olds, using a Mann–Whitney test. To estimate the least-squares means of vertical axis and vector magnitude counts for the nine behaviors, mixed models were used incorporating the within-subject random effects into the estimates. Covariance structure was determined based on Akaike information criteria (AIC). Between variance components (VC) and unstructured covariance that presented the equally smallest AIC, we chose the simpler, VC.

To identify the features that were different between “carried” and “ambulation,” sensitivity index, *d’*, was calculated for each feature. A feature importance metric was also calculated based on the random forest classifier. The feature importance metric from a set of trained decision trees was calculated from the decrease in node impurity weighted by the probability of reaching that node in a decision tree.

## 3. Results

The median age of the 21 participants (nine one-year olds) was 25 months (interquartile range: 18 to 27 months; and min and max: 13 and 35 months). The sample comprised 18 Caucasians, one Asian, one Hispanic, and one African-American. Table 1 presents the frequency and duration of behavior labels that were included in the current analysis. Although we attempted to have all participants perform each of the nine behaviors at least one time, some participants did not have data for one or more behaviors, either because they were developmentally incapable of performing those behaviors or because they refused to perform some behaviors during the trials. For example, we failed to capture the “run” behavior from four participants, all of whom were one year old.

### 3.1. Sub-Aim One

**Figure 1:**
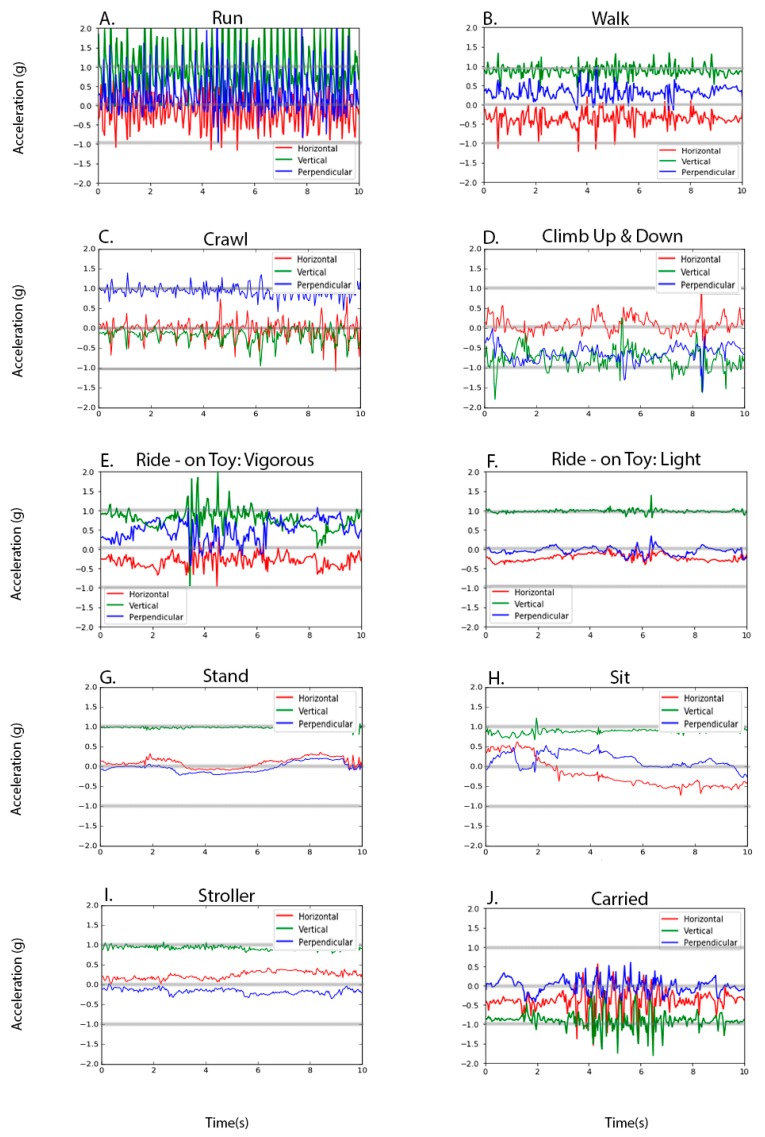
Examples of hip-worn accelerometer signals for various behaviors of toddlers.

Figure 1 illustrates examples of three-axis signals recorded by a hip-worn accelerometer during each of the nine behaviors. We observed frequent and large acceleration changes for physical activity behaviors such as “run,” “walk,” “crawl,” and “climb” (Figure 1A–D). For the “ride-on toy” behavior, we observed two distinct hip accelerometry signal patterns that reflect two different ways of riding a ride-on toy: bouncing the upper body vigorously back and force (Figure 1E) and quietly moving the feet (Figure 1F). We also observed frequent and large acceleration changes for “carried” (Figure 1J).

### 3.2. Sub-Aim Two

**Figure 2:**
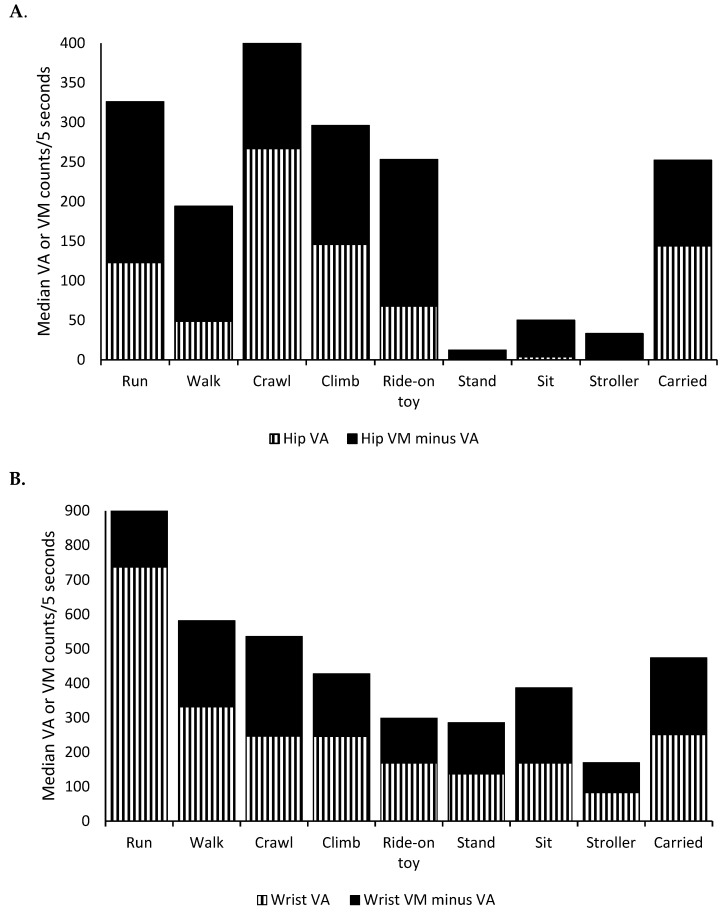
The medians of vertical axis (VA) and vector magnitude (VM) counts per 5 s for various behaviors in toddlers. (**A**) Hip-worn accelerometer counts, (**B**) wrist-worn accelerometer counts

As illustrated in Figure 2A, the median hip vertical count for “carried” (144 counts/5 s) was almost three times higher than that for “walk” (49 counts/5 s; *p* < 0.01). The median hip vertical counts for “walk” tended to be lower among one-year olds (42 counts/5 s) than two-year olds (60 counts/5 s; *p* = 0.09), although median hip vector magnitudes were not different (198 counts for one-year olds vs. 193 counts for two-year olds; *p* = 0.80). As expected, the median hip vertical count (axis 1; note the change in orientation to gravity for the “crawl” position) was high for “crawl” (267 counts/5 s) and low for “sit,” “stroller,” and “stand” (<5 counts/5 s). Although bouncing/jumping (“bounce”) was not one of the prescribed behaviors, four participants exhibited the “bounce” behavior during the trials and the “bounce” behavior presented approximately 1000 hip vertical counts/5 s (Supplemental Table S1). Further distribution statistics, including interquartile ranges, are presented in Supplemental Table S1.

In contrast to the hip data, the medians of wrist vertical axis and vector magnitude for “carried” were not higher than those for “walk” (Figure 2B). The “run” behavior presented the highest wrist vertical axis and vector magnitude counts. Although we did not quantify it, we observed in the video review process that participants were frequently holding onto something such as railings or caregiver’s hands while engaging in physical activity behaviors such as “walk” and “climb.” Some participants also frequently wiggled and tried to take off a wrist-worn monitor.

Table 2 presents the means of vertical axis and vector magnitude counts after accounting for the within-subject random effects. Based on the hip data, compared to “walk,” “run,” “crawl,” “climb,” “ride-on toy,” and “carried” exhibited higher counts, while “stand,” “sit,” and “stroller” exhibited lower counts. Based on the wrist data, compared to “walk,” “run” exhibited higher counts, while “ride-on toy,” “stand,” “sit,” and “stroller” exhibited lower counts.

*Sub-aim three.* Of the features investigated, the following features presented the highest *d’* scores to differentiate “carried” from “ambulation”: z FFT SD (*d’* = 0.64), z FFT max (*d’* = 0.61), and x FFT SD (*d’* = 0.47; Table 3). The hip accelerometer-based random forest classifier for the two behavior categories of “carried” and “ambulation” presented 89% accuracy (88% average precision and 89% average recall; F1-score of 0.88). Despite the relatively high overall accuracy, in fact the model only correctly classified 58% (31/54) of the “carried” labels, while it did so 89% (308/384) of the “ambulation” labels. This was in part due to an uneven distribution of the two classes (only 13% of behavior labels were “carried”) and a small sample size for “carried.” The feature importance of the 78 features were all lower than 0.1; the highest was vector magnitude SD (0.039), followed by y FFT median (0.034) and y FFT mean weighted (0.033).

## 4. Discussion

This study aimed to describe acceleration signal outputs from hip- and wrist-worn ActiGraph accelerometers while toddlers performed various behaviors. This investigation included a few unique behavior types in which young children engage in a free-living environment, such as “crawl,” “ride-on toy,” “stroller,” and “carried.” This study found that the median hip vertical axis count for “walk” was lower than the cut-point [14] that was proposed to define moderate- and higher-intensity physical activity (MVPA) among toddlers. “Stand,” “sit,” and “stroller” behaviors recorded <5 hip vertical axis counts/5 s. However, “carried,” a type of sedentary behavior, recorded higher vertical axis counts than “walk.” Overall, the use of hip vertical axis counts alone may be unable to capture the most frequent physical activity behavior of toddlers, “walk,” and potentially incorrectly classify “carried” as physical activity. This study suggests that the use of various acceleration signal features could help to detect and differentiate “carried” from physical activity behaviors. This study also found that although wrist vertical axis and vector magnitude counts for “carried” were not higher than those for “walk,” they were higher than the cut-points to define sedentary behavior among children age 5 years or older [23,24,25].

An interesting observation from our sub-aim two analysis was that a low vertical count (median of 49 counts/5 s) was recorded while a toddler was walking. It has been reported that at a given activity intensity level, age is inversely associated with accelerometer vertical axis counts among children [26,27]. Similarly, to define MVPA, lower vertical count cut-points have been suggested for younger children (≥419–420 counts/15 s for toddlers [14] and preschoolers [28] vs. ≥2296/60 s [574 counts/15 s] for children age 5 to 15 years) [29,30]. The current study also found a tendency towards a lower vertical count for “walk” among one-year olds compared to two-year olds. This inverse association is presumably due to a lower vertical acceleration associated with a smaller body size. The median hip vertical count for “walk” (49 counts/5 s) was lower than the cut-point proposed for MVPA among toddlers (≥419 counts/15 s [≥140 counts/5 s]) [14]. This finding implies that, based on the vertical axis count cut-points [14], the majority of walking behaviors that toddlers perform in natural play settings would be considered as light-intensity physical activity and as such would be left out of MVPA estimates. However, this does not necessarily indicate that toddlers are unable to have a high vertical acceleration, considering the high hip vertical counts recorded while performing “run,” “climb,” and “jump/bounce” behaviors. We suggest that future studies further explore if accelerometry outputs other than a vertical axis count could help to more accurately estimate physical activity, including “walk,” among toddlers.

The sub-aim two analysis revealed that “carried,” a type of sedentary behavior, recorded relatively high activity counts from both hip and wrist data. In particular, the median hip vertical count for “carried” (144 counts/5 s) was higher than the cut-point for MVPA among toddlers ≥419 counts/15 s [≥140 counts/5 s]) [14]. Also, the median wrist counts for “carried” (251 vertical axis counts/5 s and 474 vector magnitude counts/5 s) were higher than the cut-points for sedentary behavior among children age 5 years or older (<203 vertical axis counts/5 s and <397 vector magnitude counts/5 s [25] or ≤1756 vertical axis counts/minute [≤146 vertical axis counts/5 s] and ≤3958 vector magnitude counts/ minute [≤330 vector magnitude counts/5 s]) [23,24]. The acceleration signals for “carried” might partly reflect the adult’s walking movement. Misclassification of “carried” to physical activity could be of great concern since the misclassification would bias the health benefits of physical activity toward the null.

To overcome this issue, we attempted to differentiate “carried” from “ambulation” by utilizing various accelerometry signal features (sub-aim three). In ranking the quality of features for differentiation, we noted that the top 10 features included basic quantiles (e.g., min, median, and max) of single axis direct values and FFT values but did not include histogram bins. In addition, the features of vector magnitude values were ranked high, suggesting the importance of the inclusion of overall magnitude. Although the machine learning classifier developed in the current study presented relatively high overall accuracy, caution is required in interpreting the results because the analysis was not powered to develop a reliable activity recognition classifier largely due to a small sample size. The refinement and validation of a machine learning classifier with a larger sample size is warranted. It would also be an important next step to understand in what circumstances and for how much time young children are carried in everyday life, and to examine the correlation between the amount of times spent in being “carried” and in physical activity. If the time spent in the “carried” behavior is minimal, the effect of the misclassification could be ignorable. For example, in center-based childcare settings, the “carried” behavior could be minimal. If the time is significant and there is an inverse correlation between the times spent in “carried” and physical activity, the effect of the misclassification could be significant and should be appropriately treated.

There were a few characteristics to note for the other two unique behaviors of toddlers: “ride-on toy” and “stroller.” Typical “ride-on toy” behavior mostly involves lower body movement without substantial movement of the trunk or arms. Therefore, the acceleration signal patterns for “ride-on toy” could be similar to those for cycling in older children. However, in the sub-aim one analysis, we observed that some toddlers vigorous bounced their upper body to move forward, which recorded large acceleration signals. The current study confirmed in the sub-aim two analysis that the “stroller” behavior recorded low hip vertical counts, similar to “sit.” Reflecting frequent arm movements for toy play during “sit,” but not as much during “stroller,” we observed that the wrist activity counts for “stroller” were even lower than those for “sit.” This finding demonstrates that the “stroller” behavior is readily correctly classified as sedentary behavior using the vertical axis count data.

Although the feasibility of using hip and/or wrist ActiGraph accelerometers among toddlers was not the purpose of this investigation, a few observations are worth noting to direct future studies. We observed that some toddlers frequently wiggled a wrist-worn monitor and held onto railings or adult’s hands during physical activity, such as walking or climbing up/down the stairs. Considering such characteristics of toddler behaviors, wrist accelerometer data could contain more noise than hip accelerometer data in estimating physical activity among toddlers.

Several limitations of this study should be acknowledged. First, this study had a small sample size. Second, because this study only focused on nine behavior types, other behavior types were ignored. Third, despite our efforts to reflect toddlers’ sporadic activity patterns by using a five-second window size rather than a longer-window size, the five-second requirement still resulted in exclusion of many behaviors of interest performed for <5 s during the trial. Despite these limitations, this study is innovative by including unique behaviors of toddlers that were missed in previous studies. In addition, accelerometer data collected during caregiver-led play in a natural setting (indoor) rather than in a lab setting could more closely reflect those in free-living conditions. However, it should also be noted that the signals for indoor physical activity could be different from those for outdoor physical activity.

## 5. Conclusions

Hip vertical axis counts alone may be unable to capture walking as physical activity and “carried” as sedentary behavior among toddlers. Machine learning techniques that utilize additional accelerometry signal features could help to recognize behavior types of toddlers, particularly to detect “carried,” which otherwise would be classified as physical activity.

## Figures and Tables

**Table 1:** Description of behavior and accelerometer data included for analysis.

Behavior	Description	Frequency	Accelerometer Data in Seconds
		*n*	Mean (Median)
Run	Running forward from one place to the other.	20	7.5 (5.0)
Walk	Walking forward from one place to the other. Taking a few side steps to grab something, for example, was not considered as “walk.”	244	7.3 (6.0)
Crawl	Moving forward on two hands and two knees to the ground	29	6.8 (5.0)
Climb	Walking up or down the stairs or a soft foam climber	47	8.3 (8.0)
Ride-on toy	Sitting on a ride-on toy and moving forward using two feet. Sitting without moving forward or sitting and being pushed by an adult, for example, was not considered as “ride-on toy.”	40	10.3 (10.7)
Stand	Standing still without lifting a foot. Moving in place was not considered “standing”	129	6.9 (5.0)
Sit *	Sitting on the ground for play such as block play. Sitting on a ride-on toy or a slide, for example, was not considered as “sit.”	84	14.5 (9.5)
Stroller	Sitting on a stroller or wagon while it is being pushed by an adult	36	12.0 (10.5)
Carried	Being held by an adult while the adult is walking. Being held by an adult without walking was not considered as “carried.”	35	9.9 (14.0)

* Sitting on a chair would have been considered as “sit.” However, no participants sat on a chair during the trials.

**Table 2:** Least square means of vertical counts and vector magnitudes for the nine behavior types among toddlers.

	Hip Vertical Counts	Hip Vector Magnitudes	Wrist Vertical Counts	Wrist Vector Magnitudes
	Estimate ± SE	Estimate ± SE	Estimate ± SE	Estimate ± SE
Run	145 ± 14 **	352 ± 24 **	774 ± 41 **	1178 ± 62 **
Walk	52 ± 12	191 ± 21	345 ± 37	596 ± 55
Crawl	241 ± 14 **	410 ± 24 **	247 ± 42	528 ± 64
Climb	169 ± 13 **	324 ± 22 **	249 ± 38	432 ± 57 *
Ride-on toy	100 ± 13 **	297 ± 22 **	182 ± 39 **	308 ± 58 **
Stand	1 ± 12 **	19 ± 21 **	121 ± 37 **	264 ± 55 **
Sit	8 ± 12 *	65 ± 21 **	189 ± 37 **	388 ± 55 **
Stroller	7 ± 13 *	57 ± 22 **	116 ± 38 **	251 ± 57 **
Carried	149 ± 14 **	258 ± 23 *	289 ± 40	519 ± 60

The least squares means were estimated using the mixed models that accounted for within-subject random effects; * *p* < 0.05 and ** *p* < 0.01 for the mean difference test against “walk”; SE, standard error.

**Table 3:** Top 10 features ranked high in d*’* score and feature importance to differentiate “carried” vs. “ambulation”.

Rank	Feature	*D’*	Feature	Feature Importance
1	FFT SD of *z*	0.64	SD of VM	0.039
2	z FFT max of *z*	0.61	FFT median of *y*	0.034
3	FFT SD of *x*	0.47	FFT mean weighted of *y*	0.033
4	SD of *z*	0.45	FFT median of *x*	0.027
5	FFT max of *z*	0.44	FFT mean weighted of *x*	0.026
6	Kurtosis of VM	0.43	Max of VM	0.024
7	Min of *z*	0.41	FFT mean of *y*	0.021
8	Kurtosis of *y*	0.39	Min of MV	0.020
9	Min of VM	0.36	FFT mean weight of *z*	0.019
10	SD of *x*	0.33	Kurtosis of VM	0.018

FFT, fast Fourier transform; SD, standard deviation; VM, vector magnitude.

## Supplementary Materials

The following are available online at https://www.mdpi.com/1660-4601/16/14/2598/s1, Table S1: Distribution of accelerometer axis and vector magnitude counts per 5 seconds for various behavior types in toddler participants.
