# Supplementary material for: Hip and Wrist-Worn Accelerometer Data Analysis for Toddler Activities
Source: Int J Environ Res Public Health. 2019 Jul 21;16(14):2598. doi: 10.3390/ijerph16142598 (PMC6678133; doi:10.3390/ijerph16142598)
Supplement: Supplementary file 1 [file ijerph-16-02598-s001.pdf]

Supplementary

Supplemental Table 1. Distribution of accelerometer axis and vector magnitude counts per 5 seconds for various behavior types in toddler participants

			Hip		Wrist	
		N	M±SD	Median (IQR)	M±SD	Median (IQR)
Run	Axis 1	17	145±80	123 (78,192)	774±425	737 (451, 1010)
	Axis 2	17	208±77	210 (151, 250)	543±314	508 (372, 750)
	Axis 3	17	203±88	174 (132, 285)	597±320	563 (383, 796)
	VM	17	352±116	326 (285, 422)	1178±601	1092 (752, 1487)
Walk	Axis 1	21	52±37	49 (27, 63)	345±96	332 (295, 362)
	Axis 2	21	123±43	113 (92, 150)	289±79	279 (212, 354)
	Axis 3	21	125±43	118 (99, 136)	313±97	321 (229, 366)
	VM	21	191±41	194 (156, 207)	596±154	582 (479, 686)
Crawl	Axis 1	16	241±70	267 (176, 288)	247±84	247 (178, 306)
	Axis 2	16	206±107	190 (130, 255)	285±75	272 (245, 345)
	Axis 3	16	186±75	187 (121,250)	305±100	300 (256, 383)
	VM	16	410±115	402 (316, 486)	528±110	536 (463, 593)
Climb	Axis 1	19	169±81	146 (125, 230)	249±120	246 (154, 352)
	Axis 2	19	157±111	147 (64, 192)	208±111	196 (150, 230)
	Axis 3	19	160±85	138 (82, 239)	211±84	201 (147, 260)
	VM	19	324±138	296 (240, 481)	432±168	428 (332, 486)
Ride-on toy	Axis 1	19	100±84	68 (24, 187)	183±84	169 (129, 242)
	Axis 2	19	133±56	124 (95, 166)	155±77	160 (107, 214)
	Axis 3	19	202±123	189 (105, 276)	136±90	118 (91, 160)
	VM	19	297±131	253 (203, 382)	308±134	299 (178, 379)
Stand	Axis 1	21	1±1	0 (0, 1)	121±67	137 (67, 159)
	Axis 2	21	8±9	6 (2, 11)	128±87	127 (42, 194)
	Axis 3	21	13±12	8 (5, 19)	146±114	141 (44, 201)
	VM	21	19±15	12 (8, 29)	264±162	286 (112, 339)
Sit	Axis 1	21	8±11	4 (1, 10)	189±97	169 (116, 225)
	Axis 2	21	29±28	26 (9, 31)	195±131	180 (103, 239)
	Axis 3	21	44±39	31 (20, 50)	205±126	192 (117, 291)
	VM	21	65±50	50 (24, 92)	388±206	387 (237, 482)
Stroller	Axis 1	20	7±17	1 (0, 6)	116±103	83 (55, 152)
	Axis 2	20	26±43	15 (3, 26)	122±112	91 (35, 177)
	Axis 3	20	36±41	21 (8, 57)	134±132	68 (37, 219)
	VM	20	57±63	33 (24, 82)	251±204	170 (103, 389)
Carried	Axis 1	18	149±93	144 (99, 176)	289±145	251 (234, 294)
	Axis 2	18	121±68	119 (79, 161)	255±105	265 (187, 300)
	Axis 3	18	163±95	144 (99, 208)	269±114	234 (186, 352)
	VM	18	258±138	252 (140, 292)	519±194	474 (445, 626)
Bounce/jump	Axis 1	4	977±398	978 (697, 1258)	554±140	543 (439, 668)

	Axis 2	4	201±98	187 (123, 278)	530±270	542 (321, 739)
	Axis 3	4	395±182	330 (271, 519)	417±148	453 (302, 532)
	VM	4	1094±426	1070 (799, 1389)	928±291	984 (748, 1108)

M±SD, mean±standard deviation; IQR, interquartile range; VM, vector magnitude
